# An association study of the HSPA8 gene polymorphisms with schizophrenia in a Polish population

**DOI:** 10.1007/s12192-021-01249-7

**Published:** 2021-12-21

**Authors:** Malgorzata Kowalczyk, Aleksander Owczarek, Renata Suchanek-Raif, Krzysztof Kucia, Jan Kowalski

**Affiliations:** 1 Department of Medical Genetics, School of Pharmaceutical Sciences, Medical University of Silesia, Jednosci 8, 41-200 Sosnowiec, Poland; 2 Health Promotion and Obesity Management Unit, Department of Pathophysiology, Faculty of Medical Sciences in Katowice, Medical University of Silesia, Katowice, Medykow 15, 40-752 Katowice, Poland; 3 Department of Psychiatry and Psychotherapy, School of Medical Sciences, Medical University of Silesia, Katowice, Ziolowa 45, 40-635 Katowice, Poland

**Keywords:** HSPA8/HSC70, Case–control study, Single-nucleotide polymorphism, Schizophrenia, PANSS

## Abstract

Heat shock cognate 70 (HSC70/HSPA8) is considered to be a promising candidate gene for schizophrenia (SCZ) due to its many essential functions and potential neuroprotective properties in the CNS (e.g., HSC70 is involved in the turnover of the synaptic proteins, synaptic vesicle recycling, and neurotransmitter homeostasis). An alteration in the expression of *HSPA8* in SCZ has been reported. This implies that the genetic variants of *HSPA8* might contribute to schizophrenia pathogenesis. The present study attempted to determine whether *HSPA8* polymorphisms are associated with a susceptibility to schizophrenia or whether they have an impact on the clinical parameters of the disease in a Polish population. A total of 1066 participants (406 patients and 660 controls) were recruited for the study. Five SNPs of the HSPA8 gene (rs2236659, rs1136141, rs10892958, rs1461496, and rs4936770) were genotyped using TaqMan assays. There were no differences in the allele or genotype distribution in any of the SNPs in the entire sample. We also did not find any *HSPA8* haplotype-specific associations with SCZ. A gender stratification analysis revealed that an increasing risk of schizophrenia was associated with the rs1461496 genotype in females (OR: 1.68, *p* < 0.05) in the recessive model. In addition, we found novel associations between *HSPA8* SNPs (rs1136141, rs1461496, and rs10892958) and the severity of the psychiatric symptoms as measured by the PANSS. Further studies with larger samples from various ethnic groups are necessary to confirm our findings. Furthermore, studies that explore the functional contribution of the *HSPA8* variants to schizophrenia pathogenesis are also needed.

## Introduction

Schizophrenia (SCZ) is a complex neuropsychiatric disorder that is characterized by positive and negative symptoms and cognitive deficits (Owen et al. 2016; Avramopoulos 2018). Although the precise pathogenesis of SCZ is largely unknown, a strong genetic predisposition to SCZ is well-established (heritability 60–80%) (Owen et al. 2016; Dean et al. 2016). Genome-wide association (GWA) studies provide evidence for a polygenic component to the risk of SCZ involving thousands of small-effect common alleles (Owen et al. 2016; Richards et al. 2016)*.* In the largest GWAS yet conducted, 270 independent loci were found to have a significant association with the disease (Schizophrenia Working Group of the Psychiatric Genomics Consortium 2020). These associations were concentrated in genes that are involved in synaptic organization, differentiation, and transmission, which is consistent with previous GWAS and pathway analyses of the SCZ risk loci (Schizophrenia Working Group of the Psychiatric Genomics Consortium 2014; Pardiñas et al. 2018; Schijven et al. 2018).

Heat shock cognate 70 (HSC70/HSPA8) is a constitutive molecular chaperone that belongs to the large HSP70 family. HSC70 is enriched in the developing CNS and unlike other HSP70 is preferentially expressed in neurons (Brown 2007). In addition to its various housekeeping chaperone functions such as translocation*,* degradation, and proper protein folding, HSC70 participates in many of the neuroprotective mechanisms that are activated when cells are in inappropriate environments (ischemia, oxidative stress, inflammatory processes) as the brain develops (Liu et al. 2012). According to the neurodevelopmental hypothesis, a disruption of early brain development increases the risk of developing SCZ later in life (Murray et al. 2017). The critical roles and potential neuroprotective properties of HSC70 in the CNS have been extensively studied in recent years (for a review, see Stetler et al. 2010; Liu et al. 2012). For example, HSC70 is required for the optimal expression of the myelin basic protein during the differentiation of the oligodendrocytes (Aquino et al. 1998). HSC70 was also found to be involved in the turnover of the synaptic proteins and plays a prominent role in clathrin-mediated endocytosis, which is a key mechanism for synaptic vesicle recycling, which, in turn, is necessary for the maintenance of neurotransmission (Bechtold et al. 2000; Gorenberg and Chandra 2017). Strong evidence implicates disturbances of dopamine (DA), glutamate, and γ-aminobutyric acid (GABA) signaling in the pathophysiology of SCZ (Owen et al. 2016). It has been reported that via its interaction with *GABA* transporter and vesicular monoamine transporter-2 (VMAT2), HSC70 plays a crucial role in the presynaptic control of neurotransmitter homeostasis within both the GABA and DA systems (Jin et al. 2003; Requena et al. 2009). More recently, it was found that HSC70 enables the efficient coupling between DA synthesis and storage by regulating the tyrosine hydroxylase (TH) activity (Parra et al. 2016).

Taking into account the essential roles that are played by HSC70 in the CNS, we hypothesized that an abnormal expression or function of HSC70 might lead to the development of neuropsychiatric disorders including SCZ. Previous studies have identified an altered expression of *HSPA8* in SCZ patients (Föcking et al. 2015; Guan et al. 2019). Genetic variations of other HSP70 genes (*HSPA1A*, *-1B*, *-1L)* have also been associated with an increased risk of SCZ and the clinical variables of the disease (Pae et al. 2005, 2009; Kim et al. 2008; Kowalczyk et al. 2014, 2018, 2020). One study revealed a difference in the *HSPA8* rs1136141 polymorphism between first-episode psychotic schizophrenic patients and healthy participants (Bozidis et al. 2014).

The aim of this study was to examine the possible association between the *HSPA8* polymorphisms (rs2236659, rs1136141, rs10892958, rs1461496, and rs4936770) and the susceptibility to SCZ in a Caucasian Polish population. In addition, we explored the potential relationships between the *HSPA8* polymorphisms and clinical variables including the age of onset, the symptoms that were determined by the PANSS and suicidal behavior.

## Materials and methods

### Study subjects

A total of 406 unrelated schizophrenia patients were recruited from inpatients who were being treated at the Department and Clinic of Psychiatry, Medical University of Silesia in Katowice and the Neuropsychiatric Hospital in Lubliniec; newly diagnosed first-episode psychosis subjects were excluded. All of the patients fulfilled the DSM-IV-TR (Diagnostic and Statistical Manual of Mental Disorders, Fourth Edition, Text Revision) criteria for the paranoid type of schizophrenia. The final diagnosis was determined by two experienced psychiatrists based on the Structured Clinical Interview for DSM-IV Axis I Disorders, Clinical Version (SCID-I-CV, First et al. 1997). Patients with any other Axis I and Axis II disorders, neurological diseases, endocrine/autoimmune disorders, or substance dependence were excluded from the study. All of the patients were hospitalized due to an acute exacerbation of schizophrenia. The psychiatric symptoms of the patients were assessed at the time of hospital admission using the Positive and Negative Syndrome Scale (PANSS) (Kay et al. 1988), which is a 30-item rating scale that is composed of three subscales: positive (items P1-P7), negative (items N1-N7), and general psychopathology (items G1–G16). In this study, we also used the modified five-factor model of the PANSS by van der Gaag et al. (2006), which includes five dimensions: positive (P1 + P3 + G9 + P6 + P5 + G1 + G12 + G16 - N5), negative (N6 + N1 + N2 + N4 + G7 + N3 + G16 + G8 + G13 − P2), disorganized (N7 + G11 + G10 + P2 + N5 + G5 + G12 + G13 + G15 + G9), excitement (G14 + P4 + P7 + G8 + P5 + N3 + G4 + G16), and emotional (G2 + G6 + G3 + G4 + P6 + G1 + G15 + G16). Referring to our previous studies, the age of onset of schizophrenia was defined as the age at which the clear psychotic symptoms first developed. The clinical details (age of onset, positive and negative family history of SCZ, suicidal behavior) were obtained from medical records and by interviewing the patients and their family members. The general characteristics of the patients are summarized in Table 1.

**Table 1:** General characteristics of the SCZ patients

Variable	SCZ
Age, years*	42 ± 12
Gender (male/female)	243 (59.9%)/163 (40.1%)
Age of onset	24 (20–30)
Duration of the illness	14 (7–23)
PANSS-positive score	21.5 ± 5.9
PANSS-negative score	25.2 ± 6.2
PANSS-general score	43.7 ± 9.1
PANSS-total score	90.4 ± 17.8
Family history of SCZ** (yes/no)	99 (24.4%)/307 (75.6%)
Suicidal behavior (yes/no)	76 (18.7%)/330 (81.3%)

^*^Data are expressed as the means ± standard deviation or the median (lower quartile–upper quartile) for the continuous variables and *n* (%) for the categorical variables

^**^Individuals with affected first-degree or second-degree relatives

The control group consisted of 660 healthy, unrelated individuals (mean age 41 ± 9 years; 53.43% males) recruited from the volunteer blood donors at the Regional Centre of Blood Donation and Treatment in Katowice. Normal volunteers were matched to the patients for age, gender, and national origin*.* The exclusion criteria for the controls included any neurological disorders, a chronic or acute physical illness (infectious, autoimmune, or allergic diseases), current psychiatric problems, a positive family history of psychiatric disorders, a history of psychiatric medication, psychiatric hospitalization, suicide attempts, and a history of substance abuse or dependency (data were obtained through a questionnaire and interview).

All of the participants were Caucasians of Polish origin living in Upper Silesia. A written informed consent form was obtained from all of the participants before the study. The study protocol was approved by the Bioethics Committee of the Medical University of Silesia.

### SNP selection criteria

We selected five polymorphisms in the HSPA8 gene. These five SNPs included rs2236659 (in the promoter), rs1136141 (in the 5′UTR), rs10892958 (in intron 1), rs1461496 (in intron 6), and rs4936770 (in intron 8). The main selection criteria were as follows: a validated minimum 0.1 minor allelic frequency (MAF) in the European population (SNP information was retrieved from the National Center for Biotechnology Information, dbSNP, http://www.ncbi.nlm.nih.gov/SNP/), assay availability, and the potential functional significance (rs2236659 may be a causative mutation site) as confirmed by previous studies (He et al. 2010).

### DNA extraction and genotyping

Peripheral blood samples were collected in tubes containing EDTA as the anticoagulant and the genomic DNA was extracted using a QIAamp DNA Blood Mini Kit (Qiagen, Valencia, CA) according to the standard protocol. The extracted DNA was quantified spectrophotometrically using a BioPhotometer plus (Eppendorf AG, Hamburg, Germany).

Genotyping was performed using the TaqMan SNP allelic discrimination method on a CFX96 real*-*time quantitative polymerase chain reaction system (Bio-Rad) in a 96-well format. The PCR reactions were conducted in a final volume of 25 μL containing 10 ng of the genomic DNA, 12.5 μL of the TaqMan Universal PCR Master Mix (Applied Biosystems, Waltham, MA, USA), 1.25 μl of the combined primers and probes mix (Applied Biosystems), and nuclease-free water. The PCR conditions were 95 °C for 10 min followed by 40 cycles at 95 °C for 15 s and 60 °C for 1 min. Two blank controls (non-template) and three replicate quality control samples that represented the specific genotypes of each SNP were also run for each analysis. Commercially available allele-specific TaqMan primers and probes were used (Applied Biosystems). The catalog numbers for the rs2236659, rs1136141, rs10892958, rs1461496, and rs4936770 polymorphisms were C_15954880_20, C_1366867_20, C_25939924_10, C_7503670_20, and C_30053329_20, respectively.

To ensure the quality control of genotyping, approximately 5% of the samples were repeated and the concordance was 100%. Samples with missing genotypes were removed from the analysis. Furthermore, the genotype frequencies were similar to the CEU sample frequencies (available on the NCBI website).

### Statistical analysis

Statistical analysis was performed using STATISTICA 13.0 PL (StatSoft, TIBCO Inc., Palo Alto, CA, USA) and R software (R Core Team (2021), R: A language and environment for statistical computing, R Foundation for Statistical Computing, Vienna, Austria (https://www.R-project.org/)). All of the tests were two-tailed and *p* < 0.05 was considered to be statistically significant. The power of the test was at least 80%. Imputations were not done for any missing data. The nominal and ordinal variables are expressed as percentages, while the descriptive variables are expressed as the mean value ± standard deviation in the case of data with a normal distribution or as the median (lower quartile–upper quartile) in the case of data with a skewed distribution. The Hardy–Weinberg equilibrium (HWE) was examined using Fischer’s exact test to compare the actual number of genotypes with the expected number. The differences in the allele frequencies and genotype distribution between the control and SCZ group were assessed using either the chi-square (*χ*^2^) test or the Fisher exact test. The distribution of the variables was evaluated using the Shapiro–Wilk test and a quantile–quantile (Q-Q) plot and the homogeneity of the variances was assessed using the Levene test. Logistic regression was used to calculate the odds ratios (OR) and 95% confidence intervals (CI). The logistic regression models (co-dominant, dominant, recessive, over-dominant, log-additive) were also used to assess any potential association with schizophrenia risk and the best fitting models were determined using the Akaike information criterion (AIC) and Bayesian information criterion (BIC). The linkage disequilibrium (LD), haplotype analysis as well as the clinical variable models (co-dominant, dominant, recessive, over-dominant, log-additive) were performed using the *SNPAssoc* package in R. The clinical models are presented as the mean with the standard error. The two-way ANOVA (sex, genotype) with Tukey’s post hoc test was used to examine the effect of the genotypes on the clinical variables (PANSS factors, age of onset, duration of the disease).

## Results

The initial analysis showed a significant difference between males and females with respect to age (39 ± 12 vs. 46 ± 12, *p* < 0.001) in the SCZ group. There was no difference in age between males and females among the controls (41 ± 9 vs. 40 ± 8, *p* = 0.48). There was no difference in age between the patients and the controls (42 ± 12 vs. 41 ± 9, *p* = 0.21). We did find a difference in the duration of the disease and the age of onset between the male and female patients with SCZ (duration: males 12 years (7–20), females 17 years (9–25), *p* < 0.01; age of onset: males 23 years (20–27), females 27 years (22–33), *p* < 0.001). There was no difference between the SCZ male and female subgroups in the percentage of individuals with a family history of SCZ (25.1% males vs. 23.5% females, *p* = 0.71) or suicidal behavior (19.3% males vs. 17.8% females, *p* = 0.69).

### *HSPA8* genotypes/alleles and schizophrenia risk

No deviation from the Hardy–Weinberg Equilibrium was detected in either the SCZ or control groups for the five polymorphisms (SCZ: rs2236659 (*p* = 0.62), rs1136141 (*p* = 0.45), rs10892958 (*p* = 0.11), rs1461496 (*p* = 0.75), and rs4936770 (*p* = 0.60); controls: rs2236659 (*p* = 0.19), rs1136141 (*p* = 0.38), rs10892958 (*p* = 0.81), rs1461496 (*p* = 0.08), and rs4936770 (*p* = 0.15)). The genotype distributions and allele frequencies of the five studied *HSPA8* SNPs in the SCZ patients and healthy controls are summarized in Table 2. None of the SNPs significantly differed in either the genotype or allele distributions including the results of the gender-stratified analysis. We did not find any differences between the patients with a positive and a negative family history of SCZ with respect to the *HSPA8* genotype or allelic distribution.

**Table 2:** Comparison of the allele frequencies and genotype distributions of the five *HSPA8* SNPs between the SCZ cases and normal controls in all, female and male samples

dbSNP	Genotype/allele	All		*χ*^2^	*p*	Females		*χ*^2^	*p*	Males		*χ*^2^	*p*	
		SCZ	Controls			SCZ	Controls			SCZ	Controls			
rs2236659	A/A	362 (89.2)	579 (87.7)	-	0.24	150 (92.0)	273 (88.6)	-	0.45	212 (87.2)	306 (86.9)	-	0.45	
	A/G	44 (10.8)	76 (11.5)			13 (8)	33 (10.7)			31 (12.8)	43 (12.2)			
	G/G	0	5 (0.8)			0	2 (0.6)			0	3 (0.9)			
	A	768 (94.6)	1234 (93.5)	0.87	0.35	313 (96.0)	579 (94.0)	1.35	0.24	455 (93.6)	655 (93.0)	0.08	0.78	
	G	44 (5.4)	86 (6.5)			13 (4.0)	37 (6.0)			31 (6.4)	49 (7.0)			
rs1136141	G/G	290 (71.4)	472 (71.5)	0.01	0.99	117 (71.8)	232 (75.3)	-	0.32	173 (71.2)	240 (68.2)	1.28	0.53	
	G/A	104 (25.6)	169 (25.6)			40 (24.5)	71 (23.1)			64 (26.3)	98 (27.8)			
	A/A	12 (3.0)	19 (2.9)			6 (3.7)	5 (1.6)			6 (2.5)	14 (4.0)			
	G	684 (84.2)	1113 (84.3)	0	1.00	274 (84.0)	535 (86.9)	1.16	0.28	410 (84.4)	578 (82.1)	0.89	0.35	
	A	128 (15.8)	207 (15.7)			52 (16.0)	81 (13.1)			76 (15.6)	126 (17.9)			
rs1461496	G/G	155 (38.2)	254 (38.5)	2.80	0.25	57 (35.0)	102 (33.1)	5.16	0.08	98 (40.3)	152 (43.2)	0.55	0.76	
	G/A	189 (46.5)	328 (49.7)			74 (45.4)	167 (54.2)			115 (47.3)	161 (45.7)			
	A/A	62 (15.3)	78 (11.8)			32 (19.6)	39 (12.7)			30 (12.4)	39 (11.1)			
	G	499 (61.5)	836 (63.3)	0.68	0.41	188 (57.7)	371 (60.2)	0.48	0.49	311 (64.0)	465 (66.0)	0.45		0.50
	A	313 (38.5)	484 (36.7)			138 (42.3)	245 (39.8)			175 (36.0)	239 (34.0)			
rs4936770	C/C	223 (54.9)	336 (50.9)	2.53	0.28	98 (60.1)	175 (56.8)	0.60	0.74	125 (51.4)	161 (45.7)	2.60		0.27
	C/T	153 (37.7)	281 (42.6)			56 (34.4)	117 (38.0)			97 (39.9)	164 (46.6)			
	T/T	30 (7.4)	43 (6.5)			9 (5.5)	16 (5.2)			21 (8.6)	27 (7.7)			
	C	599 (73.8)	953 (72.2)	0.55	0.46	252 (77.3)	467 (75.8)	0.18	0.67	347 (71.4)	486 (69.0)	0.66		0.42
	T	213 (26.2)	367 (27.8)			74 (22.7)	149 (24.2)			139 (28.6)	218 (31.0)			
rs10892958	C/C	254 (62.6)	402 (60.9)	2.18	0.33	106 (65.0)	201 (65.3)	2.08	0.35	148 (60.9)	201 (57.1)	1.24	0.54	
	C/G	127 (31.3)	228 (34.5)			48 (29.4)	98 (31.8)			79 (32.5)	130 (36.9)			
	G/G	25 (6.2)	30 (4.5)			9 (5.5)	9 (2.9)			16 (6.6)	21 (6.0)			
	C	635 (78.2)	1032 (78.2)	0	1.00	260 (79.8)	500 (81.2)	0.19	0.66	375 (77.2)	532 (75.6)	0.32	0.57	
	G	177 (21.8)	288 (21.8)			66 (20.2)	116 (18.8)			111 (22.8)	172 (24.4)			

The genotype frequencies were further examined using the different genetic models: co-dominant, dominant, recessive, over-dominant, and log-additive. For rs1461496, there was a significant association between genotypes and increased risk of SCZ in the recessive model (OR: 1.68, 95% CI = 1.01–2.81, *p* < 0.05) in females after the gender-stratified analysis. There was also a trend toward significance in the co-dominant and over-dominant models (Table 3). Inheritance modeling did not indicate any significant differences in the distribution of the four other SNPs between the SCZ patients and the controls (data not shown).

**Table 3:** Analysis of the SNP rs1461496 based on the different inheritance models

Model	Genotype	All			Females			Males		
		SCZ/Controls *n* (%)	OR (95% CI)	*p*	SCZ/Controls *n* (%)	OR (95% CI)	*p*	SCZ/Controls *n* (%)	OR (95% CI)	*p*
Co-dominant	G/G G/A A/A	155 (38.2)/254 (38.5) 189 (46.6)/328 (49.7) 62 (15.3)/78 (11.8)	1.00 0.94 (0.72–1.23) 1.30 (0.88–1.92)	0.25	57 (35.0)/102 (33.1) 74 (45.4)/167 (54.2) 32 (19.6)/39 (12.7)	1.00 0.79 (0.52–1.21) 1.47 (0.83–2.59)	0.08	98 (40.3)/152 (43.2) 115 (47.3)/161 (45.7) 30 (12.3)/39 (11.1)	1.00 1.11 (0.78–1.57) 1.19 (0.70–2.05)	0.76
Dominant	G/G G/A-A/A	155 (38.2)/254 (38.5) 251 (61.8)/406 (61.5)	1.00 1.01 (0.79–1.31)	0.92	57 (35.0)/102 (33.1) 106 (65.0)/206 (66.9)	1.00 0.92 (0.62–1.37)	0.68	98 (40.3)/152 (43.2) 145 (59.7)/200 (56.8)	1.00 1.12 (0.81–1.57)	0.49
Recessive	G/G-G/A A/A	344 (84.7)/582(88.2) 62 (15.3)/78 (11.8)	1.00 1.34 (0.94–1.93)	0.10	131 (80.4)/269 (87.3) 32 (19.6)/39 (12.7)	1.00 **1.68 (1.01–2.81)**	** < 0.05**	213 (87.7)/313 (88.9) 30 (12.3)/39 (11.1)	1.00 1.13 (0.68–1.88)	0.64
Over-dominant	G/G-A/A G/A	217 (53.4)/332 (50.3) 189 (46.6)/328 (49.7)	1.00 0.88 (0.69–1.13)	0.32	89 (54.6)/141 (45.8) 74 (45.4)/167 (54.2)	1.00 0.70 (0.48–1.03)	0.07	128 (52.7)/191 (54.3) 115 (47.3)/161 (45.7)	1.00 1.07 (0.77–1.48)	0.70
Log-additive	–-	–-	1.09 (0.90–1.31)	0.37	–-	1.12 (0.84–1.48)	0.43	–-	1.10 (0.86–1.40)	0.46

*OR*, odds ratio; *CI*, confidence interval

### Haplotype associations

The linkage disequilibrium analysis showed a strong LD between all of the pairs of SNPs (Table 4). In view of the strong LD, only five haplotypes of the five SNPs were estimated to have a frequency of > 5%. There were no significant differences in the individual haplotype frequencies between the SCZ group and the controls in the entire sample or in the male and female subgroups as is shown in Table 5. However, for the AGGTC haplotype, there was a tendency toward statistical significance in females (*p* = 0.07).

**Table 4:** Pairwise linkage disequilibrium of the HSPA8 gene polymorphisms

SNP1	SNP2	D`	r^2^	p
rs2236659	rs1136141	0.992	0.012	< 0.001
rs2236659	rs1461496	0.996	0.038	
rs2236659	rs4936770	0.998	0.173	
rs2236659	rs10892958	0.988	0.227	
rs1136141	rs1461496	0.967	0.104	
rs1136141	rs4936770	0.967	0.407	
rs1136141	rs10892958	0.979	0.641	
rs1461496	rs4936770	0.992	0.220	
rs1461496	rs10892958	0.990	0.163	
rs4936770	rs10892958	0.990	0.732

**Table 5:** Haplotype analysis of the *HSPA8* SNPs (rs2236659–rs1136141–rs1461496–rs4936770–rs10892958) among the SCZ cases and the healthy controls

Haplotype	All			Females			Males		
	Frequency	OR (95% CI)	*p*	Frequency	OR (95% CI)	*p*	Frequency	OR (95% CI)	*p*
**A–G–A–C–C**	0.3707	1.00	–-	0.4003	1.00	–-	0.3486	1.00	–-
**A–G–G–C–C**	0.3532	0.95 (0.77–1.16)	0.59	0.3587	0.89 (0.66–1.22)	0.48	0.3479	1.04 (0.78–1.37)	0.81
**A–A–G–T–G**	0.1533	0.95 (0.72–1.24)	0.69	0.1380	1.12 (0.73–1.70)	0.60	0.1654	0.85 (0.60–1.20)	0.36
**G–G–G–T–G**	0.0603	0.80 (0.54–1.18)	0.26	0.0531	0.62 (0.32–1.22)	0.16	0.0655	0.89 (0.55–1.46)	0.65
**A–G–G–T–C**	0.0542	0.71 (0.46–1.09)	0.11	0.0426	0.48 (0.22–1.08)	0.07	0.0631	0.80 (0.47–1.38)	0.42
**Rare**	0.0083	1.77 (0.48–6.46)	0.39	0.0073	1.57 (0.31–7.97)	0.58	0.0094	1.80 (0.47–6.84)	0.39

*OR*, odds ratio; *CI*, confidence interval

### HSPA8 variants and clinical variables

A two-way ANOVA (sex × genotype) was performed to assess the association between the *HSPA8* polymorphisms (rs2236659, rs1136141, rs10892958, rs1461496, and rs4936770) and the clinical parameters: age of onset, duration of the disease, and the severity of symptoms measured by the PANSS (Table 6). There was a significant main effect of the rs1136141 genotype on a PANSS-positive score (*p* < 0.001) and the total PANSS score (*p* < 0.05) (Table 6). For a PANSS-positive score, Tukey’s post hoc test revealed significant differences between the G/G and G/A female carriers (*p* < 0.01), the G/A and A/A female carriers (*p* < 0.05), the G/G and A/A male carriers (*p* < 0.01), and the G/A and A/A male carriers (*p* < 0.01). We also observed that the females and males who carried the rs1136141 A/A genotype had lower mean scores of the positive PANSS than those with the G/A and G/G genotypes. In a five-factor model of the PANSS by van der Gaag et al. (2006), significant associations were observed between the genotypes of rs1136141 and the positive (*p* < 0.01), disorganized (*p* < 0.05), and excitement factor scores (*p* < 0.01). For the positive factor, Tukey’s post hoc test showed significant differences between the G/G and G/A female carriers, the G/G and A/A male carriers, and the G/A and A/A male carriers (all *p* < 0.01). For the excitement factor, Tukey’s post hoc test showed significant differences between the G/G and G/A female carriers (*p* < 0.01) and the G/A and A/A male carriers (*p* < 0.05). There was also a significant effect of the rs10892958 genotype on the excitement factor scores (*p* < 0.05). Tukey’s post hoc test showed significant differences between the C/C and C/G female carriers (*p* < 0.05).

**Table 6:** Results from the two-way ANOVA (sex, genotype) on the clinical characteristics of SCZ patients

	Variable	rs2236659 A/G			rs1136141 G/A			rs1461496 G/A			rs4936770 C/T			rs10892958 C/G		
		Sex	Genotype	Sex × genotype	Sex	Genotype	Sex × genotype	Sex	Genotype	Sex × genotype	Sex	Genotype	Sex × genotype	Sex	Genotype	Sex × genotype
	Age of onset	** < 0.001**	0.89	0.54	0.09	0.62	0.21	** < 0.001**	0.24	0.62	** < 0.001**	0.65	0.88	** < 0.001**	0.75	0.35
	Duration	0.21	0.76	0.67	0.07	0.73	0.38	** < 0.05**	0.41	0.29	** < 0.05**	0.96	0.46	** < 0.05**	0.77	0.70
	Total PANSS	0.13	0.98	0.69	0.99	** < 0.05**^a^	0.42	0.36	0.27	0.18	0.13	0.92	0.78	0.19	0.56	0.92
Three-factor model of PANSS	Positive	0.47	0.69	0.73	0.67	** < 0.001**^b^	0.26	0.38	0.07	0.41	0.20	0.25	0.96	0.26	0.14	0.39
	Negative	0.17	0.87	0.43	0.96	0.99	0.71	0.73	0.95	0.14	0.36	0.98	0.99	0.54	0.96	0.92
	General	0.13	0.74	0.64	0.83	0.07	0.64	0.35	0.17	0.26	0.13	0.97	0.90	0.17	0.69	0.98
Five-factor model of PANSS*	Positive	0.37	0.73	0.95	0.058	** < 0.01**^c^	0.13	0.34	0.09	0.63	0.31	0.71	0.34	0.40	0.50	0.32
	Negative	0.09	0.96	0.23	0.58	0.67	0.45	0.67	0.78	0.20	0.25	0.84	0.89	0.39	0.81	0.98
	Disorganized	0.32	0.76	0.96	0.69	** < 0.05**^d^	0.89	0.51	0.11	0.28	0.18	0.60	0.94	0.18	0.50	0.90
	Excitement	0.14	0.59	0.81	0.92	** < 0.01**^e^	0.31	0.20	0.21	0.28	0.06	0.13	0.49	0.06	** < 0.05**^f^	0.27
	Emotional	0.09	0.66	0.41	0.88	0.35	0.40	0.19	0.27	0.91	0.36	0.46	0.57	0.50	0.62	0.81

Nominally significant *p* values are bolded

^*^van der Gaag’s model

^a^Mean total PANSS scores were women—G/G 86.8 ± 18.5, G/A 92.7 ± 17.3, A/A 85.0 ± 16.2; men—G/G 91.6 ± 17.1, G/A 93.7 ± 18.7, A/A 79.0 ± 15.6

^b^Mean positive PANSS scores were women—G/G 20.3 ± 5.6, G/A 23.1 ± 5.5, A/A 18.2 ± 5.2; men—G/G 21.7 ± 5.9, G/A 22.9 ± 5.7, A/A 15.3 ± 6.2

^c^Mean positive PANSS scores were women—G/G 22.1 ± 5.7, G/A 24.6 ± 4.8, A/A 20.6 ± 6.3; men—G/G 23.7 ± 5.2, G/A 24 ± 5.4, A/A 17.8 ± 6.1

^d^Mean disorganized PANSS factor scores were women—G/G 30.9 ± 7.2, G/A 32.3 ± 7.2, A/A 28.8 ± 6.7; men—G/G 31.9 ± 6.9, G/A 33.7 ± 6.6, A/A 28.1 ± 4.2

^e^Mean excitement PANSS factor scores were women—G/G 19.1 ± 5.3, G/A 21.9 ± 6.3, A/A 18.6 ± 5.1; men—G/G 20.8 ± 5.3, G/A 22.1 ± 6.3, A/A 17.2 ± 5.6

^f^Mean excitement PANSS factor scores were women—C/C 19.1 ± 5.3, C/G 21.5 ± 6.3, G/G 18.4 ± 4.6; men—C/C 20.8 ± 5.3, C/G 21.5 ± 5.9, G/G 21.9 ± 7

We further tested the association between the *HSPA8* SNPs and the severity of the SCZ symptoms as measured using the classic three-factor model of the PANSS in five genetic models (co-dominant, dominant, recessive, over-dominant, and additive). Three SNPs were associated with the PANSS score. The genotypes of rs1136141 were associated with a PANSS-positive score based on the results of the co-dominant (*p* < 0.001 for the “A/A” genotype), dominant (*p* = 0.05 for the “A/G-G/G” genotype), recessive (*p* < 0.01 for the “A/A” genotype), and over-dominant (*p* = 0.0017 for the “G/A” genotype) models. In the gender-stratified analysis, we observed an association between a PANSS-positive score and the rs1136141 genotypes in the co-dominant (*p* < 0.01 for the “A/A” genotype), dominant (*p* < 0.05 for the “A/G-G/G” genotype), and over-dominant (*p* < 0.01 for the “G/A” genotype) models in females and co-dominant (*p* < 0.01 for the “A/A” genotype) and recessive (*p* < 0.01 for the “A/A” genotype) models in males. The rs1136141 genotypes were also associated with the total PANSS score based on the results of the co-dominant (*p* < 0.05 for the “A/A” genotype) and over-dominant (*p* < 0.05 for the “G/A” genotype) models in the entire sample. The rs1461496 genotypes were associated with a PANSS-positive score based on the results of the recessive model in the entire sample (*p* < 0.05 for the “A/A” genotype; the mean positive PANSS scores were A/A 20.5 (se 0.66) and G/G-G/A 21.74 (se 0.32)). In the gender-stratified analysis, we observed significant associations only in the male sample. The genotypes of rs1461496 were associated with a PANSS-positive score in the recessive model (*p* < 0.05 for the “A/A” genotype; the mean positive PANSS scores were A/A 19.7 (se 0.95) and G/G-G/A 22.2 (se 0.41)) and with the total PANSS score in the recessive model (*p* < 0.01 for the “A/A” genotype; the mean total PANSS scores were A/A 84.7 (se 2.8) and G/G-G/A 92.8 (se 1.2)). A strong tendency toward significance was also observed for the total PANSS score in the co-dominant model (*p* = 0.051 for the “A/A” genotype; the mean total PANSS scores were G/G 91.9 (se 1.9), G/A 93.7 (se 1.5), and A/A 84.7 (se 2.8)). In addition, we observed an association between the rs10892958 genotypes and a PANSS-positive score in the over-dominant model in females (*p* < 0.05 for the “C/G” genotype; the mean positive PANSS scores were C/G 22.29 (se 0.9) and C/C-G/G 20.3 (se 0.5)).

We also examined the potential association between suicidal behavior and individual *HSPA8* polymorphisms; however, the results were statistically insignificant.

## Discussion

This study attempted to determine whether polymorphisms in the HSPA8 gene are associated with the risk of SCZ or have an impact on the clinical parameters of the disease in a Polish population. While the direct effect of HSPA8/HSC70 on the development of schizophrenia needs to be established, there is some potential impact on the SCZ pathogenesis through the various critical functions that are performed by the HSC70 in the CNS (HSC70 is involved in the turnover of the synaptic proteins, clathrin-mediated endocytosis, neurotransmitter homeostasis, and also participates in many neuroprotective mechanisms). One of the more suggestive pieces of evidence that links HSC70 to schizophrenia is the reported change in the HSPA8/HSC70 expression in patients with SCZ compared to healthy individuals (Föcking et al. 2015; Guan et al. 2019). Several studies have also found that the popular antipsychotic drugs (haloperidol, clozapine, olanzapine, risperidone) affect the expression of the *HSPA8* gene/protein (Lauterbach 2013; Cassoli et al. 2016; Li et al. 2018), which indicates that HSPA8 may potentially be involved in the treatment of schizophrenia.

To the best of our knowledge, this is only the second study that investigated the association between the *HSPA8* polymorphisms and SCZ. However, unlike the first study of Bozidis et al. (2014), which focused on first-episode psychotic schizophrenic patients, our study group consisted of patients who were recruited during a hospitalization due to an acute exacerbation of schizophrenia while newly diagnosed first-episode psychosis subjects were excluded in order to prevent any discrepancies in the diagnosis or clinical measures. In addition, the set of SNPs that we selected was also different (only the rs1136141 variant was used in both studies), which means that four *HSPA8* polymorphisms (rs2236659, rs10892958, rs1461496, and rs4936770) were tested for their association with SCZ for the first time.

The four selected polymorphisms of *HSPA8* are located in the non-coding regions: rs10892958, rs1461496, and rs4936770 in the introns, while rs1136141 is located in the untranslated region (5′UTR). The fifth selected SNP (rs2236659) is located in the promoter of the HSPA8 gene and can affect gene expression as was shown in a previous study (He et al. 2010). To date, there have been no published papers that have evaluated the effect of the other *HSPA8* polymorphisms on gene expression and/or protein function. However, previous studies have provided evidence that SNPs in the intron regions may have an impact on either the transcriptional activity or the splicing efficiency by generating splice variants of the transcripts and disrupt the binding and function of the long non-coding RNAs, whereas SNPs in the 5′-UTR affect protein synthesis (Cooper 2010; Deng et al. 2017). Identifying the causality between specific SNPs and *HSPA8* gene regulation is undoubtedly crucial for understanding the impact of *HSPA8* on the risk of SCZ and symptomatology.

At present, we have successfully genotyped five SNPs in different regions of the HSPA8 gene. Unfortunately, we did not find any significant association of the *HSPA8* polymorphisms with the risk of SCZ in the entire study population in either the single marker or haplotype-based analysis. In contrast to our study, Bozidis et al. (2014) found a significant difference in the allelic frequency between the SCZ patients and healthy participants for the rs1136141 polymorphism with a higher incidence of the A allele carriers among the patients. There are several possible reasons for this discrepancy. First, the small number of participants in the previous study (only 50 patients and 50 healthy controls). Second, significantly different diagnostic criteria for SCZ as well as the inclusion criteria that were used for the previous research (drug-naïve patients who had been diagnosed within the schizophrenia spectrum according to the ICD-10 criteria). Third, the effects of some variants on the risk of schizophrenia might be specific to certain populations. Many genetic studies have demonstrated population-specific risk variants that are driven by underlying differences in the allele frequency, LD patterns, or gene-environmental interactions (Lam et al. 2019; Legge et al. 2021).

Among the most interesting results of our study were the gender differences in the rs1461496 genotype after examining the genotype frequencies using the different genetic models (co-dominant, dominant, recessive, over-dominant, and log-additive). The genotypes of s1461496 were associated with the risk of SCZ only in females based on the results of the recessive model (OR: 1.68, *p* < 0.05 for the A/A genotype). There was also a trend toward significance in the co-dominant and over-dominant models in females. Additionally, we observed a tendency toward statistical significance for the AGGTC haplotype in females (*p* = 0.07). The gender-specific differences in SCZ in various domains such as prevalence, symptoms, and responses to treatment have been extensively described (Li et al. 2016; Riecher-Rössler et al. 2018). Some genetic studies have suggested gender-specific differences in the etiology and pathogenesis of SCZ (Li et al. 2016). Recently, two genome-wide genotype-by-sex analyses of neuropsychiatric disorders have found that genes that have sex-dependent effects were enriched for the neuron- and synapse-related sets (Blokland et al. 2021; Martin et al. 2021). Both candidate gene studies and GWAS in SCZ have reported that the sex-specific effects were primarily found among females (Goldstein et al. 2013). Female-specific associations with SCZ have been found for a number of genes, including *RELN* (Shifman et al. 2008), *ZNF804A* (Zhang et al. 2011), *MIR137* (Yin et al. 2021), and *GALR1* (Li et al. 2020). In a previous study, we observed that sex might modulate the risk that is conferred by the *HSPA1A* rs1043618 polymorphism for SCZ as females with the rs1043618 genotype have a two-fold greater risk of developing schizophrenia than males carrying the same genotype (Kowalczyk et al. 2014). It was, therefore, not so surprising that a significant difference in the *HSPA8* genotype was found among the females with SCZ*.*

We also investigated the association between the *HSPA8* SNPs and the severity of SCZ symptoms as measured by the PANSS. We found a significant association between the s1136141 genotype and a PANSS-positive score (*p* < 0.001) and the total PANSS score (*p* < 0.05). We observed that both females and males with the rs1136141 A/A genotype had lower mean scores of positive PANSS than those with the G/A and G/G genotypes. In the five-factor model of the PANSS, significant associations were observed between the rs1136141 genotype and the positive (*p* < 0.01), disorganized (*p* < 0.05), and excitement factor scores (*p* < 0.01) with lower scores for the A/A genotype carriers for all of those factors. A less prominent association was found between the rs10892958 genotype and the excitement factor scores (*p* < 0.05). Using the five genetic models, we found that three SNPs were associated with the PANSS score. Similar to the previous ANOVA results, the rs1136141 genotypes were associated with a PANSS-positive score (in the co-dominant, dominant, recessive, and over-dominant models) and the total PANSS score (in the co-dominant and over-dominant models). A new finding was the association between the rs1461496 genotypes and the PANSS-positive scores based on the results of the recessive model in the entire sample and in the male, but not female samples, after the gender-stratified analysis. In males, the rs1461496 genotypes were also associated with the total PANSS score in the recessive model. Carriers of the homozygous rs1461496 A/A genotype had lower mean scores of the positive and total PANSS than those with the G/A-G/G genotypes. These results are interesting because the rs1461496 A/A genotype was found to increase the risk of developing SCZ in females based on the results of the recessive model. In addition, we observed an association between the rs10892958 genotypes and a PANSS-positive score in the over-dominant model in females.

It is worth noting that the *HSPA8* polymorphisms primarily influenced the positive SCZ symptoms (delusions, hallucinations, disorganized speech) in this work. Our previous studies reported a correlation between the *HSPA1A* (rs1043618) and *HSPA1B* (rs539689 and rs1061581) polymorphisms and positive symptoms in SCZ patients (Kowalczyk et al. 2014, 2020). Pae et al. (2009) also found an association between the *HSPA1B* rs539689 SNP and PANSS-positive scores at discharge in their experiment investigating the impact of the HSP70 polymorphisms on the clinical symptoms and drug response in schizophrenic patients. They speculated that HSP variations might differentially contribute to the development of negative and positive SCZ symptoms in accordance with the molecular role of these proteins. As was mentioned earlier, HSC70 may affect neurotransmitter homeostasis within the GABA and DA systems (Jin et al. 2003; Requena et al. 2009). Moreover, the uncoating of clathrin-coated vesicles by HSC70 is required for the recycling and endocytosis of the synaptic vesicles and for maintaining neurotransmission in the synapses (Gorenberg and Chandra 2017). Numerous pharmacological and brain imaging studies have demonstrated that psychotic symptoms are triggered by a dysregulation of dopaminergic neurotransmission (mainly dopamine hyperactivity in the mesolimbic dopamine pathway) in the brain (Owen et al. 2016; Stahl 2018). HSC70 might regulate dopamine neurotransmission and homeostasis in several ways*.* It has been reported that HSC70 can bind to the VMAT2 and that it negatively regulates its activity in the synaptic vesicles (Requena et al. 2009). It has also been shown that the interaction between HSC70 and TH upregulates the enzyme activity and promotes its targeting to the synaptic vesicles, which supports a role of HSC70 in the control of presynaptic dopamine homeostasis (Parra et al. 2016). A recently published study found that the dopamine D3 receptor negatively regulates the plasma membrane dopamine transporter (DAT) activity, most likely via an association of DAT with HSC70 and the stimulation of the clathrin*-*dependent internalization of the DAT (Chang et al. 2020). D3 receptors are densely expressed in the mesolimbic areas that are considered to be important for psychotic symptoms (Maramai et al. 2016). The reuptake of DA through DAT is the key mechanism for controlling the spatial and temporal dynamics of dopamine neurotransmission (Vaughan and Foster 2013).

Suicidal behavior often occurs in conjunction with schizophrenia and there were 18.7% of patients with a history of suicide attempts in our study group. Previous research has demonstrated changes in the expression of the HSPA8 protein in the amygdala and the prefrontal cortex of suicide victims (Kékesi et al. 2012). Therefore, we examined the potential association between suicidal behavior and individual *HSPA8* polymorphisms. Unfortunately, we did not find any significant association.

The results of our study are not without several notable limitations. First, the cohort size was relatively small, especially in the subgroup analyses. Therefore, the possibility of false-positive findings cannot be excluded, and therefore, the findings require replication. On the other hand, we selected a highly homogenous sample with respect to ethnicity, geographic region (Polish Caucasians from Upper Silesia), and schizophrenia subtypes (patients with a diagnosis of paranoid schizophrenia exclusively) to strengthen the confidence in our results. Moreover, the patients were assessed by the same experienced clinicians and none of the patients was newly diagnosed in order to prevent any discrepancies in the diagnosis and clinical measures. Population stratification is a major bias that can affect the genetic association results in candidate gene association studies. Second, we found that the genotypes of rs1461496 were associated with a risk of SCZ only in females based on the results of the recessive model. Thus, a further functional analysis is needed to clarify the biological mechanisms of this female-specific association between the *HSPA8* rs1461496 variant and the pathogenesis of *SCZ.* Finally, we did not investigate the effect of the *HSPA8* SNPs on the gene/protein function, which is necessary to explain the observed associations between the rs1136141, rs1461496, and rs10892958 polymorphisms and the severity of the SCZ symptoms as measured by the PANSS (both the three- and five-factor models of the PANSS were used).

In conclusion, our findings indicate that the SNPs in *HSPA8* might be associated with a higher risk of SCZ in females (s1461496) as well as the severity of the psychiatric symptoms (rs1136141, rs1461496, and rs10892958) in the Polish population. Larger studies that are based on different ethnic groups are necessary to confirm our results. Furthermore, studies that explore the functional contribution of the *HSPA8* variants to SCZ pathogenesis are also needed.

## Data Availability

All of the data used to support the findings of this study are available within the article and from the corresponding author upon reasonable request.
